# Development of a Reverse Genetics System for Toscana Virus (Lineage A)

**DOI:** 10.3390/v12040411

**Published:** 2020-04-07

**Authors:** Akira J. T. Alexander, Marie-Pierre Confort, Sophie Desloire, James I. Dunlop, Srikeerthana Kuchi, Vattipally B. Sreenu, Daniel Mair, Gavin S. Wilkie, Ana Da Silva Filipe, Benjamin Brennan, Maxime Ratinier, Frédérick Arnaud, Alain Kohl

**Affiliations:** 1 MRC-University of Glasgow Centre for Virus Research, Glasgow G61 1QH, Scotland, UK; james.dunlop@glasgow.ac.uk (J.I.D.); srikeerthana.kuchi@glasgow.ac.uk (S.K.); sreenu.vattipally@glasgow.ac.uk (V.B.S.); daniel.mair@glasgow.ac.uk (D.M.); gavin.wilkie@gmail.com (G.S.W.); ana.dasilvafilipe@glasgow.ac.uk (A.D.S.F.); ben.brennan@glasgow.ac.uk (B.B.); 2 IVPC UMR754, INRAE, Univ Lyon, Université Claude Bernard Lyon1, EPHE, PSL Research University, F-69007 Lyon, France; marie-pierre.confort@univ-lyon1.fr (M.-P.C.); sophie.desloire@univ-lyon1.fr (S.D.); maxime.ratinier@univ-lyon1.fr (M.R.)

**Keywords:** reverse genetics, *Phlebovirus*, *Bunyavirales*, Toscana virus

## Abstract

Toscana virus (TOSV) is a *Phlebovirus* in the *Phenuiviridae* family, order *Bunyavirales*, found in the countries surrounding the Mediterranean. TOSV is an important cause of seasonal acute meningitis and encephalitis within its range. Here, we determined the full sequence of the TOSV strain 1500590, a lineage A virus obtained from an infected patient (Marseille, 2007) and used this in combination with other sequence information to construct functional cDNA plasmids encoding the viral L, M, and S antigenomic sequences under the control of the T7 RNA promoter to recover recombinant viruses. Importantly, resequencing identified two single nucleotide changes to a TOSV reference genome, which, when corrected, restored functionality to the polymerase L and made it possible to recover infectious recombinant TOSV (rTOSV) from cDNA, as well as establish a minigenome system. Using reverse genetics, we produced an NSs-deletant rTOSV and also obtained viruses expressing reporter genes instead of NSs. The availability of such a system assists investigating questions that require genetic manipulation of the viral genome, such as investigations into replication and tropism, and beyond these fundamental aspects, also the development of novel vaccine design strategies.

## 1. Introduction

Toscana virus (TOSV) belongs to the genus *Phlebovirus* of the *Phenuiviridae* family (order *Bunyavirales*), first isolated in 1971 from *Phlebotomus perniciosus* and *P. perfiliewi* sand flies in Italy [1,2]. This and related viruses are widely spread around the Mediterranean basin [3]. Members of the *Phlebovirus* genus are characterised by their enveloped, trisegmented, negative-sense single-stranded RNA genome. The L segment encodes for the RNA dependent RNA polymerase (RdRp). The M segment encodes the surface glycoprotein precursors Gn and Gc, and a non-structural protein NSm within a single NSm/Gn/Gc open reading frame. The S segment is ambisense and has the nucleocapsid protein N in the negative sense and the second non-structural protein NSs in the positive sense [4,5,6,7]. TOSV is prevalent in the countries surrounding the Mediterranean, as well as islands within, with three distinct co-circulating lineages, i.e., A, B, and C, which roughly correspond to geographical location with lineage A toward the east and B toward the west. Lineage C appears to be present in Croatia and Greece, although it has never been isolated. The lineages can co-circulate, and both lineage A and B are found in France and Turkey [8,9,10,11,12,13,14,15,16,17]. TOSV is indeed widespread, with detection reported in North African countries (reviewed in [8]), and also more recently detection in Bulgaria [18].

TOSV is found in both the male and female sand flies in the wild, and after artificial infection, can be sexually transmitted between adults and transovarially transmitted to larvae. Seroprevalence appears high among domestic animals and dogs in particular. This could play a role in maintaining levels of TOSV while the sand fly numbers are reduced during the winter months [1,19,20,21,22,23,24,25].

Seroprevalence among humans is around 10% to 24%, occasionally reaching 40% within the range of sand fly distribution; suggesting rates of asymptomatic or mild infection is relatively high and seroprevalence increases with age, steadily increasing in age groups over 10 years (thus suggesting exposure time to sand flies is important in transmission and infection) [14,16,18,26,27,28,29,30,31]. Although mild cases of TOSV infection generally are self-limiting febrile illnesses that require no treatment, TOSV exhibits a tropism for the central nervous system (CNS) and cases in the areas around the Mediterranean, as well as in people who holiday in the Mediterranean area, have been described. The outcome of infection is usually good and there is no recurrence of symptoms. The virus, however, has the potential to cause severe aseptic meningitis, meningoencephalitis, and severe infections beyond the currently known geographical range of the virus [32,33,34,35,36,37,38,39,40,41,42,43,44]. Keeping preparedness in focus, it is important to develop tools to study such viruses.

Studies of TOSV has been limited due to the absence of rescue systems to generate recombinant virus, thus not allowing for direct genetic manipulation of the virus. Here, we present the development of a minigenome system, as well as a reverse genetics system for TOSV lineage A and show that genetic manipulation could be achieved. We generated viruses no longer expressing the type I interferon antagonist NSs [45], either deleted or replaced with reporter genes. These novel tools could assist studies on this emerging pathogen by allowing direct manipulation of the virus.

## 2. Materials and Methods

### 2.1. Cell Culture

Cells used were A549, A549 NPro (expressing BVDV NPro; a kind gift of R. Randall, University of St. Andrews), BSR, and BSRT7/5 CL21 [46] (a clone based on BSRT7/5 cells; obtained from K.-K. Conzelmann, Ludwig-Maxmilians-Universität München, Germany [47]). The A549 and A549 NPro cells were grown in DMEM (Gibco, Thermo Fisher Scientific, Waltham, MA, USA) supplemented with 10% fetal bovine serum (FBS) and 100 units/mL penicillin and 100 µg/mL streptomycin with the A549 NPro cells additionally supplemented with blasticidin at 10 µg/mL. BSR and BSRT7/5 CL21 cells were grown in GMEM (Gibco, Thermo Fisher Scientific, Waltham, MA, USA) supplemented with 10% FBS, 10% tryptose phosphate broth (TPB), pencillin/streptomycin with the BSRT7/5 CL21 cells additionally supplemented with 0.25 mg/mL G418. All cell culture was carried out at 37 °C and 5% CO_2_.

### 2.2. Viral Rescue

All transfections were done using TransIT^®^-LT1 Transfection Reagent (Mirus, Madison, WI, USA) according to the manufacturer’s instructions. The BSRT7/5 CL21 cells were seeded at a density of 3 × 10^5^ cells per well in a six-well plate. After 24 h, and at 80% confluency, cells were transfected with 500 ng of each plasmid containing the antigenomic S, M, and L segments under control of the T7 RNA polymerase promoter. The plates were kept at 37 °C and 5% CO_2_ until the first sign of CPE, or 6 days. Then, the media was collected and clarified by centrifugation. Supernatants designed as passage 0 (P0) stocks were stored at −80 °C.

### 2.3. Viral Culture and Stocks

TOSV (strain 1500590, lineage A, [12]) was inoculated at a low multiplicity of infection (MOI) (0.001), or 100 µL of P0 BSRT7/5 CL21 rescue supernatant per 20 mL, into A549 NPro cells in DMEM supplemented with 2% FBS. Cells were grown at 33 °C and media harvested once CPE began to occur or after 2 weeks, whichever was earlier. The media was clarified by centrifugation and stocks frozen at −80 °C.

### 2.4. Rescued Viral Culture and Stocks

To create stocks of rTOSV, the media from 6-day-old transfected BSRT7/5 CL21 cells was clarified by centrifugation before 100 μL was added to a 150 cm^2^ flask of A549 NPro cells at 60% confluency in 2% FCS containing DMEM. The flask was incubated at 33 °C, 5% CO_2_, for 7 days, until the cells began to show signs of CPE. The media was removed and clarified by centrifugation before freezing at −80 °C.

### 2.5. Virus Titration

The virus was titred by plaque assay on A549 NPro cells under an overlay of 1× MEM (Gibco, Thermo Fisher Scientific, Waltham, MA, USA), 2% newborn calf serum (NBCS) and 0.6% Avicel. These were incubated at 37 °C and 5% CO_2_, for 3 to 11 days. Then, cells were fixed in 4% formaldehyde and stained with trypan blue for visualisation. All viral titres referred to are given as plaque forming units/mL (PFU/mL).

### 2.6. Minigenome Replication Assay

A *Renilla* luciferase reporter plasmid, pTOSV hRen, was designed by substituting the M segment coding region with a negative-sense, humanised *Renilla* luciferase ORF under the control of the T7 RNA polymerase promoter, and synthesised (Genscript, Piscataway, NJ, USA). This was transfected alongside a Firefly luciferase transfection control, pTM1-FFLuc, which was used for normalization of the *Renilla* luciferase output. The reporter was used in combination with the L and N expression plasmids, pTM1-TOSV-L and pTM1-TOSV-N. Unless otherwise described, the plasmids were used at 500 ng each, transfected into 3 × 10^5^ BSRT7/5 CL21 cells in 6-well plates using TransIT^®^-LT1. The luciferase expression was measured from cell lysate after 48 h, using the Promega Dual-Luciferase^®^ Reporter Assay System.

### 2.7. Nanoluciferase (NLuc) Expression Assay

Cells were infected with rTOSV ΔNSs:NLuc at the MOI stated in the text. To measure NLuc expression, the cells were lysed in Promega passive Lysis buffer before the lysate was mixed 1:1 with Promega (Madison, WI, USA) Nano-Glo Luciferase Assay Substrate. The reactions were allowed to equilibrate for 10 min at room temperature before measurement.

### 2.8. Viral Growth Curves

The A549 NPro cells were seeded at a density of 3 × 10^5^ in six-well plates. The virus was added at a MOI of 0.01 per well. After one hour (37 °C, 5% CO_2_), virus containing media was removed and the well washed in PBS. After washing, 2 mL of 10% FCS containing DMEM (Gibco, Thermo Fisher Scientific, Waltham, MA, USA) was added and removed at the appropriate time point. The media was frozen at −80 °C until titration.

### 2.9. Viral RNA Extraction, cDNA Synthesis, and RACE Analysis

The virus was grown on A549 NPro cells, as previously described, and the supernatant was collected and clarified by centrifugation. The viral stock was concentrated 10:1 using a 100 kDa NMWL protein filter. Viral RNA (stocks contain vRNA and cRNA) extraction was performed on the concentrate using the Qiagen (Hilden, Germany) QIAamp Viral RNA kit according to the manufacturer’s protocol. The RNA was polyadenylated using the Ambion (Austin, TX, USA) polyA tailing kit according to the manufacturers protocol, and cleaned using the Qiagen (Hilden, Germany) RNeasy Kit, RNA cleanup protocol. cDNA was then synthesised using SuperscriptIII Reverse Transcriptase and primer Oligo-d(T)v (GACCACGCGTATCGATGTCGACTTTTTTTTTTTTTTTTv, with v being a variable nucleotide). The 3′ termini of the S segment genome and antigenome were amplified from the cDNA using primers TOSV_RACE_S1 (CAAAGTGGCTGCCTAGTCC) or TOSV_RACE_S2 (CCTTAGCCCAAAAGGTGG), with primer RACE_AP (GACCACGCGTATCGATGTCGAC). The 3′ termini of the M segment genome and antigenome were amplified using the primers TOSV_RACE_M1 (GGTATAAGCTCCATTCCCTGG) or TOSV_RACE_M2 (CCAGAGCCTCGTAAAGGC), with the RACE_AP primer. The 3′ termini of the L segment genome and antigenome were amplified using the primers TOSV_RACE_L1 (CAGTCGACTTGTGATCCTCTG) or TOSV_RACE_L2 (GGGTCTTCATATCCATATGGG), with the RACE_AP primer. The L ORF was also amplified in overlapping 1 kb sections from cDNA to consolidate sequencing data. Fusion PCR was used to join overlapping sections into 2kb products which were, in turn, used to create two 4 kb sections covering the entire ORF. Each individual PCR reaction was sequenced to provide multiple coverage.

### 2.10. Viral RNA Extraction and RNA Sequencing

The virus was grown in large volume cell culture flasks (5 × 225 cm^2^ flasks, 150 mL media) on A549 NPro cells as in Section 2.3, and the supernatant was collected and clarified by centrifugation. The viral stock was concentrated to a final volume of 20 mL using a 100 kDa NMWL protein filter. Then, the viral particles were pelleted by ultracentrifugation at 25,000 rpm, 4 °C, for 2 h. Then, the viral RNA was extracted using a Qiagen (Hilden, Germany) RNEasy kit according to the manufacturer’s instructions, including on column DNA digestion. QC of the extracted RNA with Qubit (Thermo Fisher Scientific, Waltham, MA, USA) DNA and RNA high sensitivity assays showed that residual DNA was still present. To minimise the amount of DNA carried over into sequencing, the sample was subjected to an additional DNase treatment. Two technical replicate sequencing libraries were prepared from the RNA sample using a TruSeq stranded RNA kit, according to the manufacturer’s instructions. Then, the DNA sequencing libraries were pooled together and sequenced on a MiSeq using a v2 300 cycle Micro kit for a total of around 10 million reads. Following this, the paired end fastq files were subjected to quality control using FastQC [48], before adaptor trimming using Cutadapt [49]. All available complete nucleotide sequences relating to TOSV were downloaded from NCBI [50] and 2,856,292 paired end reads retained after QC and preprocessing were mapped to the downloaded viral nucleotide sequences using Bowtie2 aligner [51]. Then, 1,390,120 viral mapped reads were used to perform denovo assembly using SPADES genome assembler [52] using different K-mer lengths. The scaffolds were manually assembled into the L, M, and S segments. The final assembly was compared with the nucleotide collection database using BLAST [53]. The assembled sequences (complemented with RACE data) were deposited in GenBank with accession numbers MT032308, MT032307, MT032306 for L, M, and S segments, respectively. The raw transcriptome data sequenced from two TOSV samples used for the assembly are available in NCBI (Bioproject PRJNA604562, Biosample SAMN13974122, Library 1 (2 paired end sequencing files) SRR11015404 and Library 2 (2 paired end sequencing files) SRR11015403).

### 2.11. NSs Deletion Plasmids

In order to create antigenome expression plasmids with NSs deleted, the pUC57-TOSV-S plasmid was linearised by PCR using the primers S_NLuc_Fw and S_NLuc_Rv, which include the N mRNA termination signal adding 5′ overhangs with homology to NLuc. NLuc was amplified in the negative sense from a Zika virus NLuc-encoding cDNA clone [54] using the primers NLuc_Fw and NLuc_Rv (Appendix B, Table A5) adding 5′ homology to the S segment. Then, the plasmid pUC57-TOSV-S ΔNSs:NLuc was assembled from the fragments using InPhusionHD (Clontech, Mountain View, CA, USA). The NSs gene was also replaced with two fluorescent reporter genes, mCherry and mRuby, using the primers S_FP_Fw and S_FP_Rv to amplify the pUC57-TOSV-S backbone and FP_Fw and FP_Rv to amplify mCherry and mRuby (Appendix B, Table A5), generating pUC57-TOSV-S ΔNSs:mCherry and pUC57-TOSV-S ΔNSs:mRuby. In order to delete the entire NSs ORF, the N mRNA termination signal was joined directly to the 3′ antigenome UTR by amplification of pUC57-TOSV-S using primers S_ΔNSs_Fw and S_ΔNSs_Rv (Appendix B, Table A5) before reassembly, as described previously.

### 2.12. Western Blotting

Cells were lysed in passive lysis buffer and samples reduced before being run on 4% to 12% gradient BIS-TRIS polyacrylamide gels. Anti TOSV-N-MA29010 was used at a final dilution of 1:2000 and incubated overnight at 4 °C. A loading control of anti-tubulin was used at the same concentration.

The TOSV-N antibody was made by Eurogentec (Liège, Belgium) and was raised against the peptide sequence VKERGTAKGRDWKKD, amino acids 44 to 58 in the N terminus of the N sequence. This sequence, whilst exposed on a single N protein monomer, was possibly hidden by the suspected helical arrangement of N protein in its intracellular state [55]. The antibody unfortunately was non-conclusive in immunofluorescence experiments but did, however, detect the N protein in Western blots.

### 2.13. Microscopy and Image Analysis

Images were captured using an EVOS (Thermo Fisher Scientific, Waltham, MA, USA) Fl imaging system and viewed using FIJI imageJ (imagej.net/Fiji).

### 2.14. Statistical Analysis

Statistical analysis was performed using Minitab 19 (Minitab, State College, PA, USA).

### 2.15. Data Availability

Data underlying figures with luciferase readings and virus titres can be found under DOI 10.5525/gla.researchdata.967

## 3. Results and Discussion

### 3.1. Replication of a TOSV Minigenome and Viral Rescue Require Correction of the L Sequence

Initially, three plasmids based on the TOSV reference genome sequences (NC_006319.1, NC_006318.1, and NC_006320.1) in combination with some sequencing information for TOSV 1500590 were constructed. These plasmids consisted of antigenomic L, M, and S segments followed by the hepatitis delta virus (HDV) ribozyme and under control of the T7 RNA polymerase promoter and terminator: pUC57-TOSV-L, pUC57-TOSV-M and pUC57-TOSV-S. Two helper plasmids, expressing the L and the nucleocapsid N proteins in pTM1 were also constructed. The plasmids expressed an internal ribosome entry site (IRES) followed by the ORF, under control of the T7 RNA polymerase promoter and terminator: pTM1-TOSV-L and pTM1-TOSV-N. These plasmids, alongside BSRT7/5 CL21 cells, which constitutively express T7 RNA polymerase, comprised the reverse genetics system based on the previously published BUNV protocol [56]. The minigenome plasmid contained the untranslated regions (UTR) of the M segment flanking an anti-sense, humanised *Renilla* luciferase ORF followed by the HDV ribozyme and T7 terminator, again under the control of the T7 RNA promoter called pTOSV hRen. When transfected alongside the helper L and N plasmids, the luciferase protein can only be expressed if the L protein replicates and transcribes the minigenome RNA following correct encapsidation by N. Unfortunately, the minigenome failed to function and repeated attempts using different combinations and quantities of plasmid also failed to produce any recoverable virus.

**Figure 1:**
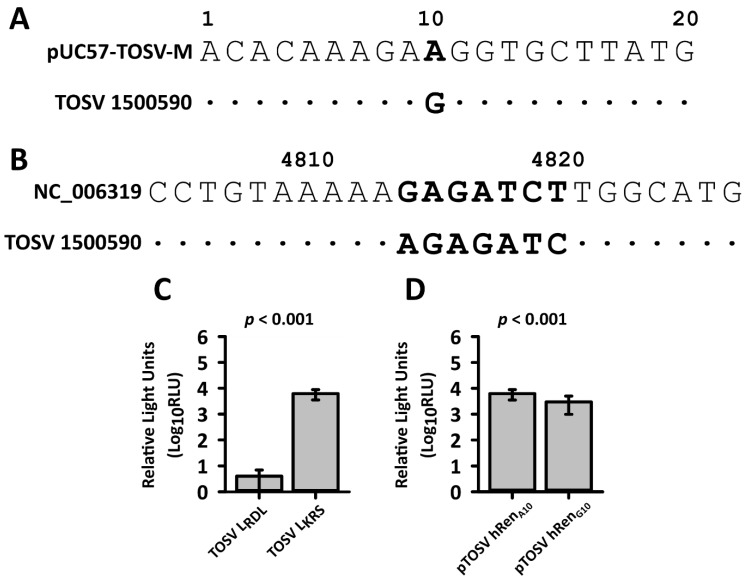
RNA sequencing of TOSV revealed critical errors in published TOSV sequences; regions in bold and (**A**) and (**B**) indicate sequence changes. (**A**) RACE analysis of TOSV identified a change at position 10 of the 5′ untranslated regions (UTR) of the M segment. As this was the segment on which the minigenome was based, this was introduced in the *Renilla* luciferase reporter plasmid to create pTOSV hRen_G10_; (**B**) Two critical changes in the L segment from that of GenBank submission NC_006319.1 were identified. This sequence only appears in this GenBank submission and not in other available sequences (see Table 1); (**C**) Replication of minigenome pTOSV hRen. After corrections to the L expression plasmid (TOSV L_KRS_) minigenome replication was observed, *N* = 16; (**D**) G at position 10 of the 5′ antigenome UTR reduced replication in the minigenome replication system, *N* = 6. Two sample t test, error bars show standard deviations. Assays shown in (**C**) and (**D**) are representative of three independent experiments. RLU, relative light units.

We subsequently reassessed viral sequences. Initially, it was thought that the problem could lie within the UTRs sequences of the minigenome as a single incorrect nucleotide can quench the replicative ability of the system [57,58]. Rapid amplification of cDNA ends (RACE) analysis was carried out on viral RNAs (from TOSV lineage A strain 1500590) and a mismatch was identified in the 5′ UTR of the antigenomic M segment sequence, on which the M rescue plasmid was based (Figure 1A). The virus contains a G at position 10 of the antigenome 5′ UTR, whereas the M-based rescue plasmid contains an A. The corresponding position to A at position 10 in the minigenome plasmid (with the transcribed minigenome RNA in genome orientation) was also changed to a G to make pTOSV hRen_G10_. In parallel, to investigate whether mutations in the sequences led to failure to rescue the virus, the entire L ORF was amplified in short overlapping 1 kb sections which were in turn assembled using multiple sequential overlap extension PCR reactions. Then, all the resulting fragments and the entire segment were sequenced. Moreover, the genome of TOSV 1500590 was also resequenced by RNA sequencing and assembled as described in Materials and Methods and combined with the RACE data to obtain up to date sequences for the entire L, M, and S segments (some relevant sequence variations are shown in Appendix A Table A1, Table A2, Table A3 and Table A4). Importantly, these approaches identified two single nucleotide (nt) changes in the ORF between the plasmid-encoded L clones, and that of the virus, i.e., a missing A at position 4814 and an extra T at position 4820. This changed three amino acids from KRS (as present in the virus) to RDL (as present in the plasmid) at amino acids 1599 to 1601 of the L protein (Figure 1B). Importantly, the KRS sequence at this location is highly conserved amongst TOSV isolates and appears to be highly conserved amongst phleboviruses as a whole, Table 1.

Once this sequence was corrected in pTM1-L, *Renilla* luciferase activity could be determined in the minigenome assay, and thus this sequence change appeared to be critical for L activity (Figure 1C), and *Renilla* activity was observed with both versions of the minigenome (Figure 1D). Indeed, the original minigenome (pTOSV hRen) and that matching the viral UTR (pTOSV hRen_G10_) were both efficiently replicated, although the latter showed a small (though significant) decrease in activity. As the A to G swap is not seen in any of the other available TOSV M segment sequences, it was not corrected in the rescue plasmid to keep as genetic marker for successful virus rescue.

### 3.2. Rescue of Recombinant TOSV from cDNA

**Figure 2:**
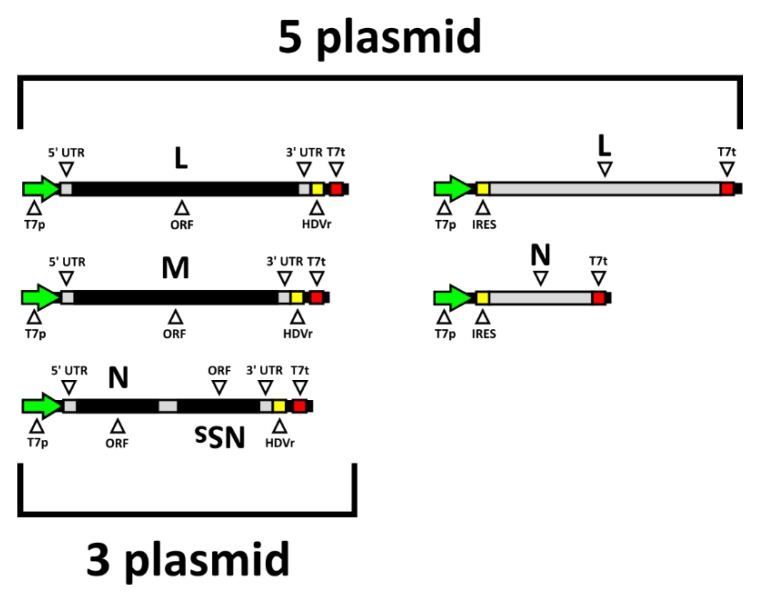
The TOSV rescue system. In the 5-plasmid system, the antigenome is expressed coupled to the hepatitis delta virus ribozyme (HDVr) for efficient RNA cleavage under control of the T7 RNA polymerase promoter (T7p) and T7 RNA polymerase terminator (T7t). These are supplemented with the L (RNA-dependent RNA polymerase, RdRp) and N (nucleocapsid) proteins. The 3-plasmid system omits these helper plasmids and relies solely on the antigenome-encoding plasmids.

To reassess the virus rescue experiments, pUC57-TOSV-L and pTM1-TOSV-L plasmids were corrected to pUC57-TOSV-L_KRS_ and pTM1-TOSV-L_KRS,_ respectively, to provide a functioning L protein. Then, 500 ng of each of the antigenome plasmids plus 500 ng of the helper plasmids (pTM1-TOSV-L_KRS_ and pTM1-TOSV-N) were transfected into BSRT7/5 CL21 cells in a six-well plate for a five-plasmid rescue. This was repeated without the two helper plasmids to test virus rescue in a the three-plasmid rescue system; a graphical representation of the rescue systems is shown in Figure 2. After six days the media was harvested, clarified by centrifugation, and then plaqued on A549 NPro cells. These initial tests indicated that the three-plasmid system was much more efficient than the five, producing titres reaching 10^6^ and 10^4^ PFU/mL, respectively. The rescue was repeated three separate times to ensure reproducibility, with each producing similar results. The three-plasmid system was, therefore, used for further TOSV rescue experiments.

**Figure 3:**
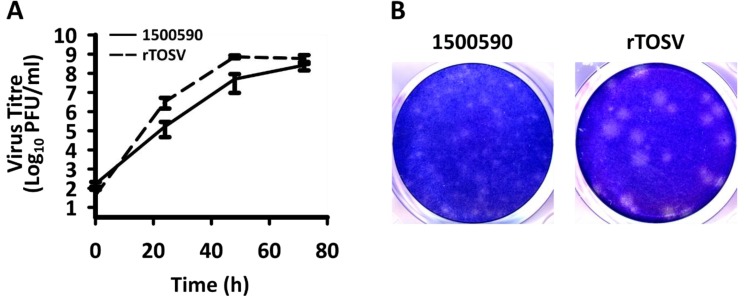
Rescue of TOSV. (**A**) rTOSV (virus rescued by three plasmid system) replication as compared with TOSV 1500590 on A549 NPro cells. The timecourse was repeated twice independently with similar results, *N* = 3. Error bars show standard deviations; (**B**) Plaque morphology of TOSV 1500590 (left panel) and rTOSV (right panel) on A549 NPro cells after 72 h.

Then, the stock was titrated by plaque assay on A549 NPro cells and the plaques were of a similar morphology, although larger compared with TOSV strain 1500590. The rescued virus (rTOSV) replicated to high titres and slightly faster than the TOSV strain 1500590 (Figure 3). This could be due to the observed change in position 10 of the M segment, which could affect virus production (see Figure 1D for the observations with the minigenome system); or other changes in rescued virus as compared with strain 1500590. Further work will be required to investigate this question.

### 3.3. Rescue of Recombinant TOSV with the NSs Protein Replaced or Deleted

**Figure 4:**
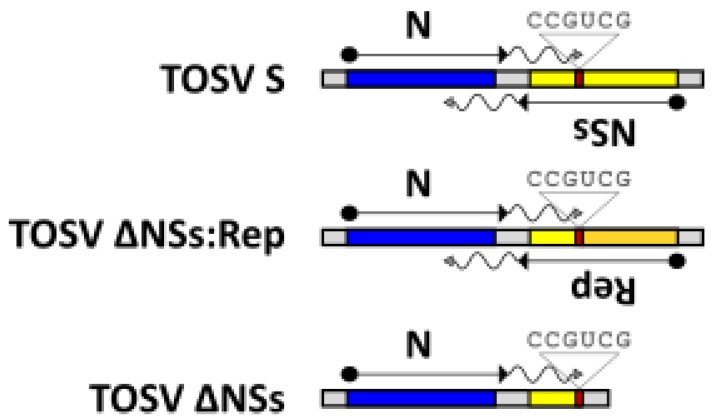
The rTOSV NSs deletion mutants. The rTOSV with NSs removed was generated from NSs deletion plasmids which replaced or deleted the NSs ORF (yellow) whilst retaining the N mRNA termination signal (CCGUCG). N ORF is represented in blue. The N mRNA termination signal resides within the NSs ORF. Reporter (Rep) (orange box) constructs where the NSs ORF has been replaced by a reporter gene retain a short C-terminal peptide of NSs containing the N mRNA termination signal through to the authentic NSs stop codon. For the ΔNSs construct, the NSs stop codon and the N mRNA termination signal were maintained, but the remainder of the ORF was deleted.

NSs proteins of phleboviruses have been shown to antagonise innate immune responses, and thus are virulence factors [45], and TOSV NSs has indeed been shown to interact with the antiviral type I interferon response [59,60,61], as an E3 ubiquitin ligase inducing Rig-I degradation [62], as well as reducing PKR levels [63,64]. When the TOSV NSs ORF is swapped into the Rift Valley fever virus MP-12 strain, the neuroinvasiveness of MP-12 was increased in mice [65], suggesting that this protein plays a role in the tropism or propagation of TOSV. Previous studies have identified that the N protein mRNA terminates within the NSs ORF and the termination signal is important for efficient viral replication, and the TOSV termination signal has been mapped previously as the sequence 3′ CCGUCG 5′ in genome RNA [66]. As NSs is non-essential for replication the rescue system was further validated by targeting this open reading frame for deletion or replacement, Figure 4.

**Figure 5:**
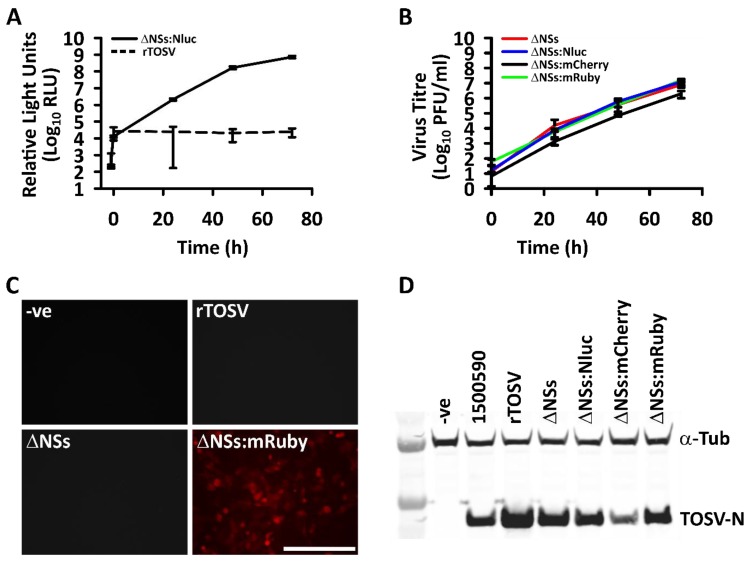
Generation of TOSV NSs deletion mutants. (**A**) Replacement of the NSs ORF with NLuc allows for measurements of viral replication within the A549 NPro cells. rTOSV is shown as a baseline control to ensure the light readings reflect NLuc activity from the replicating, reporter gene expressing virus. RLU, relative light units. The luciferase assay was repeated twice independently, *N* = 3; (**B**) Replication of TOSV NSs deletion/replacement mutants in A549 NPro cells (MOI of 0.1). Each time course was repeated twice independently, *N* = 3; (**C**) When NSs was replaced with mRuby, infected cells were clearly visible in the red fluorescence channel (mCherry is identical to mRuby), the scale bar is 200 µm; (**D**) Western blot detection of N protein in BSR cells after 48 h (MOI 0.1), tubulin detection with anti α-tubulin to show loading control. Error bars show standard deviations.

As proof of principle and in order to create the first deletion, the NSs ORF was replaced with NLuc. Then, this was used in the rescue system to generate rTOSV ΔNSs:NLuc. Following this, NLuc expression was measured over time in A549 NPro cells and activity was indeed detected (Figure 5A). The NSs gene was also replaced with two fluorescent reporter genes; mCherry and mRuby and the rescued viruses expressed mCherry and mRuby in A549 NPro cells (Figure 5C).

Finally, the entire NSs ORF was deleted and rTOSV ΔNSs was rescued. All the NSs-deletant TOSV were found to grow on A549 NPro cells (Figure 5B). The successful deletion of the NSs protein was confirmed by generating cDNA generated from the rescued virus. The S segment was amplified from the cDNA and the product sequenced. The sequences confirmed deletion of the nucleotides between 898 and 1814 of the S segment of the TOSV antigenome, and removal of most of the NSs sequence whilst retaining the mRNA termination signal of N protein.

In summary, here we present the first system for generating infectious lineage A TOSV using a T7 RNA polymerase driven, plasmid-based system containing antigenomic sequences in BSRT7/5 CL21 cells. The system provides the ability to rescue the virus from three antigenome segments encoding cDNA. We have shown that it is possible to generate recombinant viruses with altered and deleted S segments, by successfully replacing and removing the open reading frame of the virulence factor NSs. This system provides the ability to study the role(s) of proteins such as NSs. However, investigations into other elements in the TOSV genome are also now possible, as well as vaccine design for attenuated viruses. The reverse genetics system described, in this study, opens new possibilities to understand this emerging pathogen.

## Figures and Tables

**Table 1:** L protein sequences of Toscana virus (TOSV) (amino acid positions 1599 to 1601) and sequences of related phleboviruses in this region.

	Consensus	P	V	K	K	R	S	G	M
SFNV	HM566172	·	·	·	·	·	·	·	·
	HM566167	·	·	R	·	·	·	·	·
TOSV	NC_006319	·	·	·	R	D	L	·	·
	MK422498	·	·	·	·	·	·	·	·
	KU925899	·	·	·	·	·	·	·	·
	KU204977	·	·	·	·	·	·	·	·
	EF656363	·	·	·	·	·	·	·	·
	KU935735	·	·	·	·	·	·	·	·
	KU904265	·	·	·	·	·	·	·	·
	KX010934	·	·	·	·	·	·	·	·
	KU922127	·	·	·	·	·	·	·	·
	KC776216	·	·	·	·	·	·	·	·
	KU204980	·	·	·	·	·	·	·	·
	KU573067	·	·	·	·	·	·	·	·
	JX867534	·	·	·	·	·	·	·	·
	KU935736	·	·	·	·	·	·	·	·
SFSV	KM042102	·	·	·	·	·	·	·	L
SFTV	NC_015412	·	·	·	·	·	·	·	L
	GQ847513	·	·	·	·	·	·	·	L
	GQ847513	·	·	·	·	·	·	·	L
RVFV	NC_014397	·	·	·	·	·	·	·	V

Indicated are GenBank accession numbers for each virus. Abbreviations: SFNV, sand fly fever Naples virus; TOSV, Toscana virus; SFSV, sand fly fever Sicilian virus; SFTV sand fly fever Turkey virus; and RVFV, Rift Valley fever virus.

## Appendix A

Table A1, Table A2, Table A3 and Table A4 show, as indicated, selected relevant nucleotide and amino acid changes between L, M, and S segments of TOSV reference sequences and sequences of TOSV 1500590, and of rescue plasmids.

**Table A1:** RNA sequencing of L revealed a single insertion and deletion in the TOSV reference sequence at 4814 to 4820 in the GenBank TOSV sequence NC_006319 when compared with TOSV 1500590, changing the protein sequence.

	Position										4814						4820								
NC_006319		C	C	T	G	T	A	A	A	A	A	G	A	G	A	T	C	T	T	G	G	C	A	T	G
	Translation		P			V			K			R			D			L			G			M	
TOSV 1500590		·	·	·	·	·	·	·	·	·	·	A	G	A	G	A	T	C	·	·	·	·	·	·	·
	Translation		P			V			K			K			R			S			G			M	
											↑							↑							
											A ins							T del							

**Table A2:** A single nucleotide change was identified in L at position 5416 in the rescue plasmid when compared with both the TOSV reference sequence NC_006319 and TOSV 1500590.

	Position	5411												5423		
NC_006319		T	G	G	A	T	G	T	A	T	G	G	T	T	T	C
	Translation		W			M			Y			G			F	
TOSV 1500590		·	·	·	·	·	·	·	·	·	·	·	·	·	·	·
	Translation		W			M			Y			G			F	
pUC57-TOSV-L		·	·	·	·	·	·	C	·	·	·	·	·	·	·	·
	Translation		W			M			H			G			F	

**Table A3:** Changes were identified in the 5′ antigenomic UTRs of TOSV 1500590 when compared with the reference sequences.

	Position	1								10										20	
L	NC_006319	A	C	A	C	A	G	A	G	A	G	G	C	C	C	A	A	A	T	A	T
	pUC57-TOSV-L	·	·	·	·	·	A	·	·	·	·	·	·	·	·	·	·	·	·	·	·
	TOSV 1500590	·	·	·	·	·	A	·	·	·	·	·	·	·	·	·	·	·	·	·	·
M	NC_006320	A	C	A	C	A	G	A	G	A	A	G	G	T	G	C	T	T	A	T	G
	pUC57-TOSV-M	·	·	·	·	·	A	·	·	·	·	·	·	·	·	·	·	·	·	·	·
	TOSV 1500590	·	·	·	·	·	A	·	·	·	G	·	·	·	·	·	·	·	·	·	·
S	NC_006318	A	C	A	C	A	G	A	G	A	T	T	C	C	C	G	T	G	T	A	T
	pUC57-TOSV-S	·	·	·	·	·	·	·	·	·	·	·	·	·	·	·	·	·	·	·	·
	TOSV 1500590	·	·	·	·	·	·	·	·	·	·	·	·	·	·	·	·	·	·	·	·

**Table A4:** Changes were identified in the 3′ antigenomic UTRs of TOSV 1500590 when compared with the reference sequences.

	Position	40									30										20										10										1
L	NC_006319	T	A	T	A	T	C	T	A	T	A	A	G	T	T	A	T	T	T	A	A	G	A	A	T	T	G	G	G	C	G	G	T	C	T	T	T	G	T	G	T
	pUC57-TOSV-L	·	·	·	·	·	·	·	·	·	·	·	·	·	·	·	·	·	·	·	·	·	·	·	·	·	·	·	·	·	·	·	·	·	·	·	·	·	·	·	·
	TOSV 1500590	·	·	·	·	·	·	·	·	·	·	·	·	·	·	·	·	·	·	·	·	·	·	·	·	·	·	·	·	·	·	·	·	·	·	·	·	·	·	·	·
M	NC_006320	A	C	A	T	A	T	T	T	T	G	T	T	T	T	G	T	T	C	T	T	T	A	A	A	G	C	A	C	C	G	G	T	C	T	T	T	G	T	G	T
	pUC57-TOSV-M	·	·	·	·	·	·	·	·	·	·	·	·	·	·	·	·	·	·	·	·	·	·	·	·	·	·	·	·	·	·	·	·	·	·	·	·	·	·	·	·
	TOSV 1500590	·	·	·	·	·	·	·	·	·	·	·	·	·	·	·	·	·	·	·	·	·	·	·	·	·	·	·	·	·	·	·	·	·	·	·	·	·	·	·	·
S	NC_006318	A	T	T	A	C	T	A	G	C	T	C	T	G	G	T	T	T	A	G	C	A	A	T	A	C	G	G	G	A	G	G	T	C	T	T	T	G	T	G	T
	pUC57-TOSV-S	·	·	·	·	T	·	·	·	·	·	·	·	·	·	·	·	·	·	·	·	·	·	·	·	·	·	·	·	·	·	·	·	·	·	·	·	·	·	·	·
	TOSV 1500590	·	·	·	·	T	·	·	·	·	·	·	·	·	·	·	·	·	·	·	·	·	·	·	·	·	·	·	·	·	·	·	·	·	·	·	·	·	·	·	·

## Appendix B

Table A5 shows primer sequences used for this study.

**Table A5:** Primer sequences.

**S_NLuc_Fw**	aagacCATGGCTGTCTAGAAGTCTATTATTAGCTC
**S_NLuc_Rv**	aCTGGCGTGGCTGCCTAGTCCCCCC
**Nluc_Fw**	aagaccatGGCTGTCTAGAAGTCTATTATTAGCTC
**Nluc_Rv**	agacagccATGGTCTTCACACTCGAAGATTTC
**S_FP_Fw**	ctcaccatGGCTGTCTAGAAGTCTATTATTAGCTC
**S_FP_Rv**	gtacaagTGGCTGCCTAGTCCCCCC
**FP_Fw**	ctcaccatGGCTGTCTAGAAGTCTATTATTAGCTC
**FP_Rv**	agacagccATGGTGAGCAAGGGCGAG
**S_ΔNSs_Fw**	ctaggcagccAGGCTGTCTAGAAGTCTATTATTAGCTCTG
**S_ΔNSs_Rv**	tagacagcctGGCTGCCTAGTCCCCCCC
